# Implementation of digital optical phase conjugation with embedded calibration and phase rectification

**DOI:** 10.1038/s41598-018-38326-4

**Published:** 2019-02-07

**Authors:** Zhipeng Yu, Meiyun Xia, Huanhao Li, Tianting Zhong, Fangyuan Zhao, Hao Deng, Zihao Li, Deyu Li, Daifa Wang, Puxiang Lai

**Affiliations:** 1 Department of Biomedical Engineering, Hong Kong Polytechnic University, Hong Kong, Hong Kong; 2 Shenzhen Research Institute, Hong Kong Polytechnic University, Shenzhen, 518057 China; 3 School of Biological Science and Medical Engineering, Beihang University, Beijing, 100083 China; 4 Beijing Advanced Innovation Centre for Biomedical Engineering, Beihang University, Beijing, 100083 China

## Abstract

Focused and controllable optical delivery beyond the optical diffusion limit in biological tissue has been desired for long yet considered challenging. Digital optical phase conjugation (DOPC) has been proven promising to tackle this challenge. Its broad applications, however, have been hindered by the system’s complexity and rigorous requirements, such as the optical beam quality, the pixel match between the wavefront sensor and wavefront modulator, as well as the flatness of the modulator’s active region. In this paper, we present a plain yet reliable DOPC setup with an embedded four-phase, non-iterative approach that can rapidly compensate for the wavefront modulator’s surface curvature, together with a non-phase-shifting in-line holography method for optical phase conjugation in the absence of an electro-optic modulator (EOM). In experiment, with the proposed setup the peak-to-background ratio (PBR) of optical focusing through a standard ground glass in experiment can be improved from 460 up to 23,000, while the full width at half maximum (FWHM) of the focal spot can be reduced from 50 down to 10 μm. The focusing efficiency, as measured by the value of PBR, reaches nearly 56.5% of the theoretical value. Such a plain yet efficient implementation, if further engineered, may potentially boost DOPC suitable for broader applications.

## Introduction

Manipulating and focusing light deep inside biological tissue and tissue-like scattering media has been desired for long yet considered challenging due to the strong scattering of light in biological tissues^1–3^. But recent research has shown that the seemingly random scattering and the resultant speckle patterns are actually deterministic within a certain temporal window^4^. This finding has inspired a few techniques^5–8^ that aim to reverse or compensate for the scattering-induced phase distortions within scattering media. Optical phase conjugation (OPC) is one such approach that can phase conjugate (also referred to time reverse) the scattered light back to the scattering medium, making diffused photons propagating along different optical paths interfere constructively at the targeted position and hence form an optical focus behind or within the scattering sample. Initially, the phase conjugation is enabled through a 2-step (first record and then playback) holographic process in nonlinear photorefractive materials^9–13^. Despite of their advantages in responding speed, system simplicity, and building cost^9,10^, such analogue schemes usually result in limited attainable optical energy in the phase conjugated light, since the holographic playback simultaneously erases the hologram recorded in the photorefractive material^9,10,14^. Moreover, the lack of freedom to manipulate the recorded hologram pattern and the phase conjugated light, which are highly preferable in many biomedical applications^15,16^, further hinders its applications.

To address the aforementioned limitations, digital OPC (DOPC) was recently proposed by using a digital camera-spatial light modulator (SLM) aligned module for wavefront recording and wavefront playback^17–23^. In DOPC, the hologram recording and reading are physically separated—the former at the camera and the latter at the SLM—and can be arbitrarily controlled independently. The power of the phase conjugated light increases proportionally to the power of the reference light, and theoretically there is no boundary except for the damage threshold of the SLM. Furthermore, such a system allows for further manipulation of the optical wavefront prior to playback, and the response time of digital devices (cameras and SLM) can be tuned (limited by the minimum response time of the device), so the experiment time can be adjusted arbitrarily for different purposes. These flexibilities have facilitated combination of DOPC with various modulating mechanisms, and have inspired a series of approaches that are able to achieve reliable optical focusing inside or through scattering media, such as time-reversed ultrasonically encoded (TRUE) optical focusing^18,24^, time reversal of variance-encoded (TROVE) optical focusing^15^, time-reversed adapted-perturbation (TRAP)^25^ or time reversal by analysis of changing wavefronts from kinetic targets (TRACK) optical focusing^26^, as well as magnetically controlled perturbation-guided optical focusing^27,28^.

The utility and extension of DOPC systems, however, have been practically throttled largely due to two critical requirements in system design and operation^29^. First, achieving accurate pixel-to-pixel match between the digital camera and the SLM is complicated and experience demanding; the camera and the SLM should be conjugated exactly with each other, and the mismatch must be limited within one pixel^25^ in six dimensions, i.e., the three displacement axes (${\rm{\Delta }}x,\,{\rm{\Delta }}y,\,{\rm{\Delta }}z$) and the three angle axes (${\rm{\Delta }}{\beta }_{x},\,{\rm{\Delta }}{\beta }_{y},\,{\rm{\Delta }}{\beta }_{z}$)^29^. In addition, the flatness of SLM surface poses substantial influence to the focusing performance, such as the peak-to-background ratio (PBR) and the full-width of half maximum (FWHM) of the focal spot^29^. These factors need to be tuned carefully in order to achieving robust performance, for which iterative calibration methods^29,30^ have been proposed by Jang *et al*. and Azimipour *et al*., respectively. The implementation of these two methods, however, is time consuming (several minutes or even longer) as hundreds or even thousands of iterations are needed, and this calibration time length increases proportionally to the number of independently-calibrated element on the SLM. Moreover, during the experiment when the system or the environment alters (e.g. strong air flow or accidental bumping to the optical table), the effect of the sought curvature compensation, and hence the focusing performance, may reduce considerably. In this case, an extended recalibration is inevitable, which interrupts or further slows down the experiment. On the other hand, to record the phase wavefront of the sample beam, an in-line or off-axis phase-shifting holography are needed^25,31^. In the in-line setting, an electro-optic modulator (EOM) is used to execute a four-phase digital holographic method to retrieve phase^25^. Note that using the EOM requires an accurate pure phase modulation without amplitude modulation in the context, posing an extra burden to the optical system which has already been quite complicated for many researchers. In the off-axis setting, the distance between the digital camera and the SLM needs to be quite large in order to spatially separate the original and conjugated beams. The off-axis angle limitation brings extra complexity to the alignment, and the extended optical path length increases the system instability. In addition, some high frequency information may be lost due to the low utilization levels of the spatial bandwidth^32,33^.

To address the aforementioned limitations, we present a plain yet reliable DOPC setup with an embedded four-phase, non-iterative approach that can rapidly compensate for the wavefront modulator’s surface curvature. Due to the lack of an EOM at hand, a non-phase-shifting in-line holography method is developed for effective phase retrieval, which further simplifies the system design and reduces the cost. Experimentally, optical focusing with a PBR of up to 23,000 has been obtained through a standard ground glass diffuser, with a FWHM focal spot of 10 μm. The focusing efficiency, as measured by the PBR, reaches nearly 56.5% of the theoretical value.

## Methods

**Figure 1:**
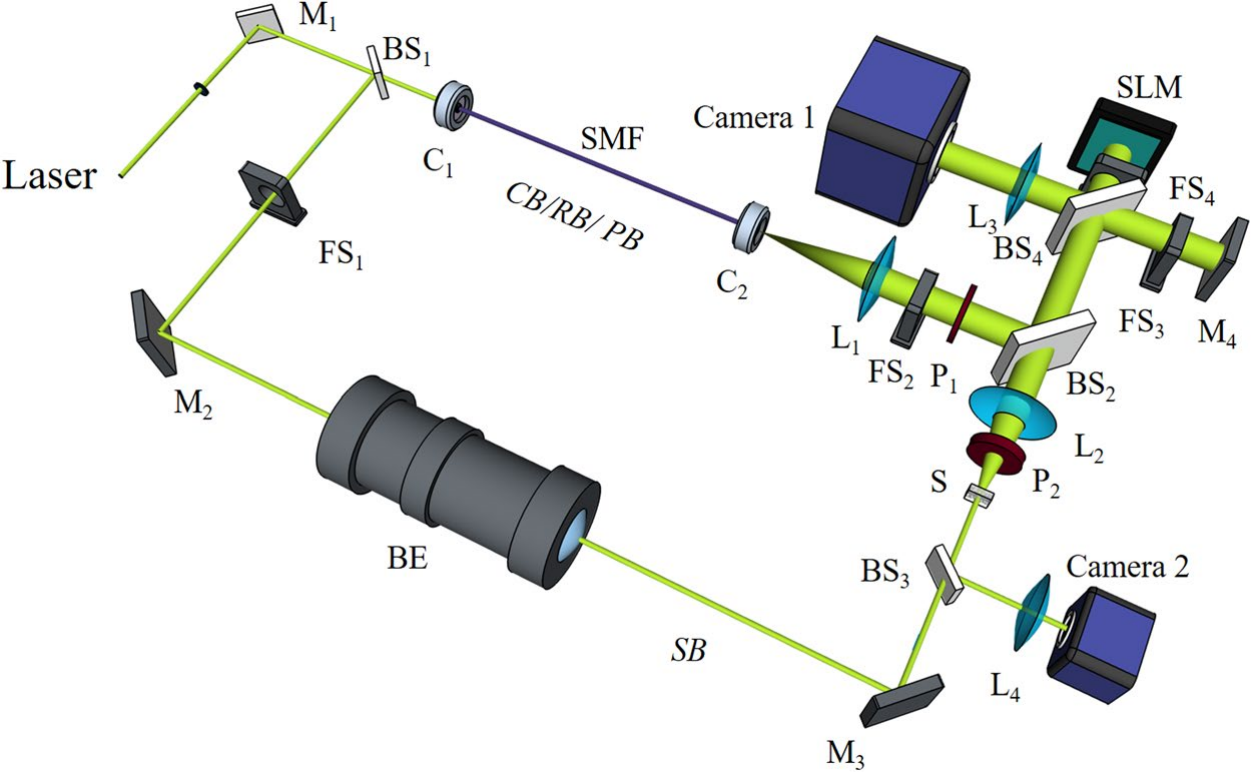
Schematic of the DOPC system. CB/RB/PB/SB: calibration/reference /playback/sample beam; BE: beam expander; BS_1_, BS_2_: cube beam splitter; BS_2_, BS_4_: plate beam splitter; C_1_, C_2_: fiber port connector; Camera 1: scientific CMOS camera; Camera 2: CMOS camera; FS_1_-FS_4_: fast shutter; HWP: half-wave plate; L_1_, L_2_, L_4_: Plano-convex lens; L_3_: camera lens; M_1–4_: mirror; PBS: polarized beam splitter; P_1,2_: polarizer; S: scattering medium; SLM: spatial light modulator; SMF: single mode fiber.

The experimental setup is shown in Fig. 1. A 532 nm continuous laser (EXLSR-532-200-CDRH, Spectra Physics) is used as the light source. Its coherence length is measured to be 300 m^34^. The laser output is split into two beams, a sample beam and a multipurpose beam (as the calibration beam, reference beam, and playback beam) by a beam splitter (BS_1_). A scientific CMOS camera (Camera 1, pco.edge 5.5, PCO) is used to image the diffused sample light exiting the scattering medium. A spatial light modulator (SLM, PLUTO-VIS-056, HOLOEYE) is positioned to conjugate to Camera 1 and phase modulate the playback beam. In additional to the abovementioned components, a single-mode fiber (SMF) and lens L_1_ are used to shape the reference beam to be planar (25.4 mm in diameter), lens L_2_ is used to collect light exiting the scattering medium and adjust the speckle grain size in Camera 1, and lens L_3_ is positioned in front of Camera 1 to image the surface of the SLM or Mirror M_1_. Four fast shutters (FS_1–4_) are used to control the on and off of different optical beams. Another camera (Camera 2) is used to observe the time-reversed playback beam. The polarization of the reference beam is adjusted by one polarizer (P_1_) to match the polarization of the SLM. The polarization of the sample beam is adjusted by another polarizer (P_2_) to get maximal interference intensity between the sample beam and the reference beam.

**Figure 2:**
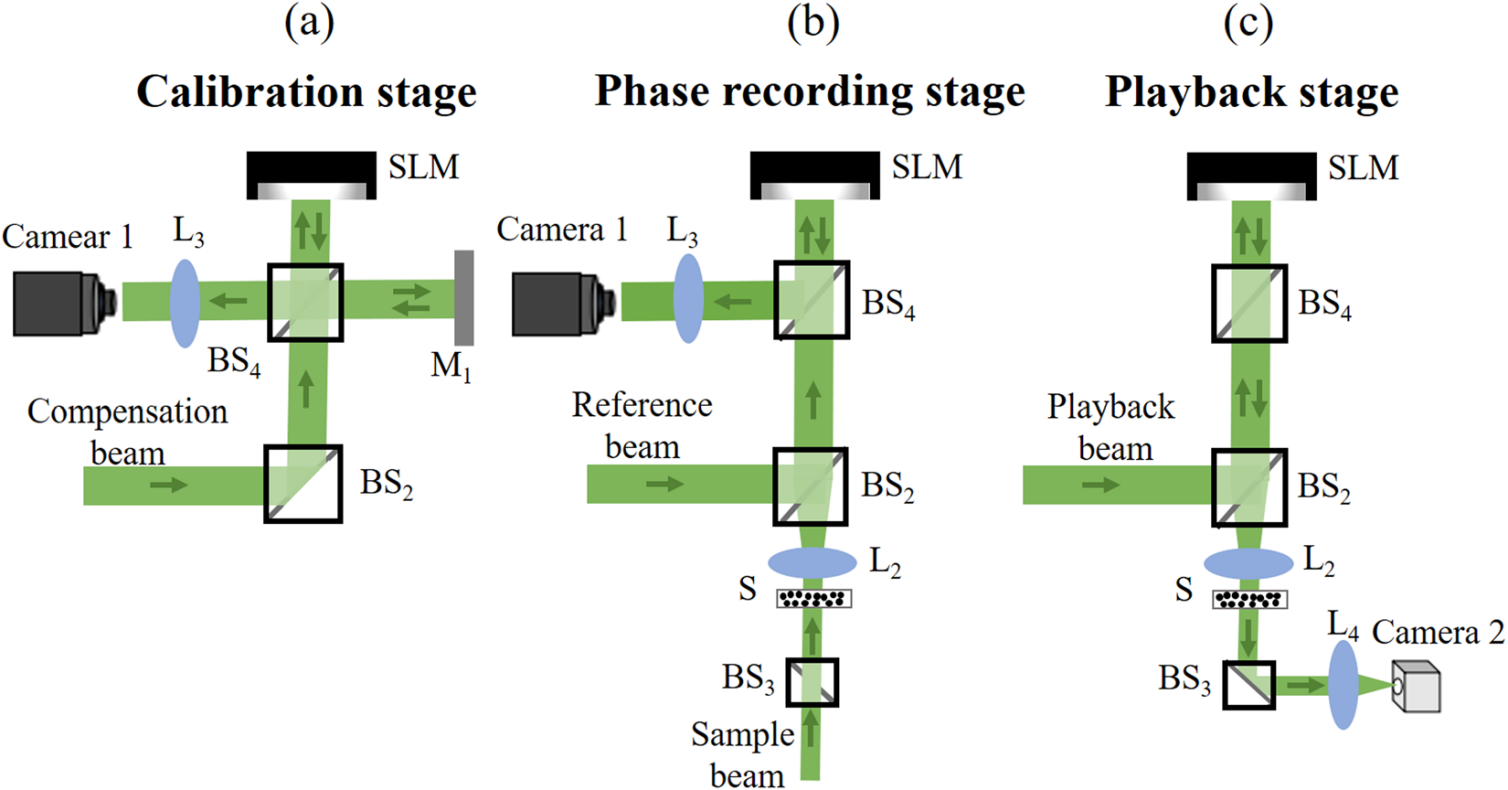
Illustration of the calibration stage (**a**), the phase recording stage (**b**), and the playback stage (**c**).

In experiment, a complete DOPC operation is divided into three stages (Fig. 2): the embedded calibration stage, the phase recording stage, and the playback stage, which are described in detail below.

**Figure 3:**
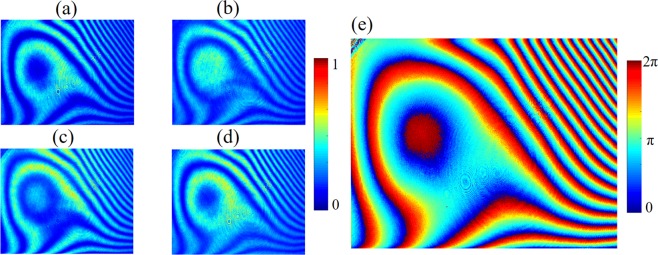
Four interferograms are recorded, when the SLM is displayed with four uniform patterns with phase angles at (**a**) 0, (**b**) π/2, (**c**) π, and (**d**) 3π/2, respectively. (**e**) The computed system compensation phase pattern corresponding to the four interferograms.

As in ref.^25^, Camera 1 and the SLM are first aligned carefully to achieve a pixel-to-pixel mismatch of less than one pixel (Fig. 2a). After that, calibration of the reference beam imperfection and the SLM surface curvature are carried out, for which no extra optical paths are required. The SLM and a mirror (M_1_) are adjusted to be completely perpendicular to the calibration beam by using a retroreflector. The calibration beam is thus reflected by the SLM and M_1_, respectively, and the reflected beams interfere in Camera 1, with interference pattern recorded by the camera. Then the SLM is displayed with four uniform patterns with phase angle at 0, π/2, π, and 3π/2, respectively^35^. The corresponding interferograms are recorded and denoted as *I*_*k*_ (*k* = 1, 2, 3, 4), respectively (Fig. 3a–d). The four interferograms can compose a complex interferogram (*CI*)^25^. The *CI* and the system compensation phase pattern (*CPP*) that has taken into account the reference beam imperfection and the SLM curvature can be expressed by

1 $${CI}=({I}_{1}-{I}_{3})+{\rm{i}}({I}_{2}-{I}_{4})$$

2 $$C{PP}={\arg }\,[{Im}({CI})/{Re}({CI})]$$

where *arg* [] denotes taking the argument. The *CPP* can be obtained from the Eqs 1 and 2 and is shown in Fig. 3e. In experiment, this compensation pattern is implanted into the DOPC system, i.e., being added to the SLM pattern obtained from regular phase retrieval procedure.

The phase retrieval procedure spans the phase recording stage and the playback stage, as shown in Fig. 2b,c. In the phase recording stage, the sample beam is expanded and illuminates the front surface of the scattering medium. Distorted sample light exiting the medium is collected and relayed Camera 1, where it interferes with the reference beam. The interfering patterns are transferred to the computer to compute the optical field, whose phase conjugation is then transferred to the SLM. In the playback stage, the sample beam is blocked, and the playback beam illuminates the SLM, generating a phase conjugated copy of the original sample beam, which travels back to the scattering medium and converges to the position of incidence at the front surface of the first scattering sample.

In the phase recording stage, the incident light field can be denoted as *E*_*in*_ and the scattering light field at Camera 1 plane as *U*. The relationship between *E*_*in*_ and *U* can be expressed by

3 $${U}={T}{E}_{{\rm{in}}}$$

where *T* is the transmission matrix of the scattering medium. In the playback stage, the light field at the SLM plane is modulated to U^*^ (* represents complex conjugate), and light leaving the SLM (*E*_*out*_) can be expressed by^25,27^

4 $${E}_{{out}}={T}^{t}{U}^{\ast }={({T}^{\dagger }T{E}_{{in}})}^{\ast }\approx {E}_{{in}}^{\ast }$$

where *t* represents transposition, and $\dagger$ represents complex conjugate transpose. Approximately, ${T}^{\dagger }T\approx I$ (*I* is the identity matrix), assuming the system is time invariant during the whole process. As seen, the output light field conjugates to the original incident light field.

**Figure 4:**
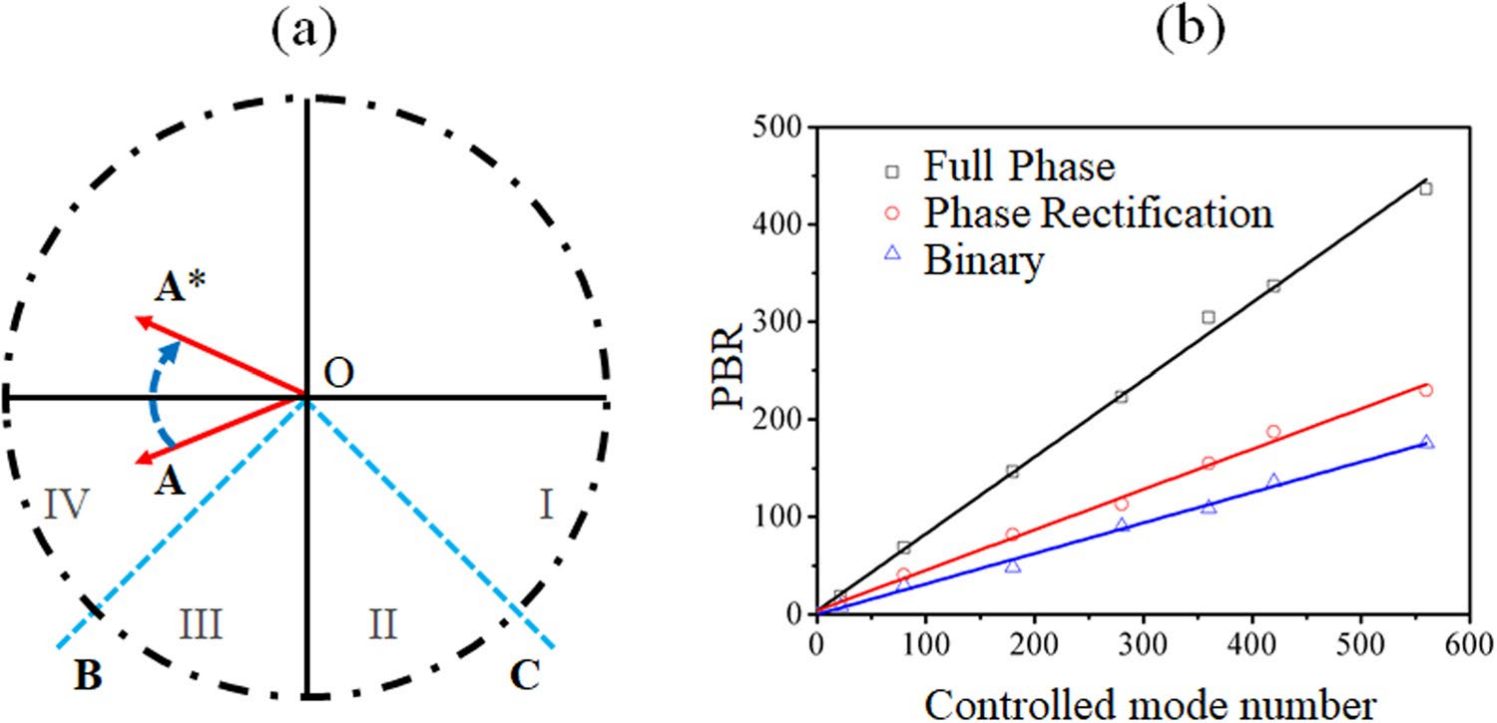
(**a**) Illustration of the phase rectification-based DOPC using wave vector decomposition. (**b**) The relationship between the theoretical PBR in DOPC and the controlled SLM pixel number using the full phase, phase rectification, and binary modulations, respectively.

In the proposed non-phase-shifting in-line holography DOPC, the sample beam, the reference beam, as well as their interferogram are one-by-one recorded by Camera 1 as *I*_*s*_, *I*_*r*_, and *I*_*int*_, respectively. They are related by

5 $${I}_{{int}}={I}_{r}+{I}_{s}+{\rm{2}}\sqrt{{I}_{r}{I}_{s}}\,\cos \,\theta $$

where *θ* is the phase difference between the reference beam and the sample beam. What should be noticed is that, according to Eq. 5, the phase information cannot be retrieved. The light field *U* can thus be expressed by ${\rm{U}}={a}+{\rm{i}}\cdot b$ (a and b are real matrixes). Full-wave rectification of ***U*** is executed, as shown in Fig. 4(a), producing a new light field *Ù*:

6 $$U^{\prime} ={\rm{a}}+{\rm{i}}\ast |b|$$

The performance of such a phase rectification-based DOPC is illustrated in Fig. 4. As shown, OB and OC are the angle bisectors of the third and fourth quadrant, respectively, which equally divide the third and fourth quadrant into four sections, marked as Region I, II, III and IV. Assuming an original wave vector ${\rm{OA}}=|{\rm{a}}|\cdot \exp (i{\rm{\alpha }})$, where $|{\rm{a}}|$ is the amplitude of the vector and ${\rm{\pi }} < {\rm{\alpha }} < 2{\rm{\pi }}$ is the phase of the vector. After being rectified, the vector becomes ${{\rm{OA}}}^{\ast }=|{\rm{a}}|\cdot \exp [{i}\cdot (2{\rm{\pi }} \mbox{-} {\rm{\alpha }})]$. Projecting vector OA^*^ onto OA, the value of the corresponding component is ${\rm{P}}=|a|\,\cdot \,\cos (2{\rm{\pi }} \mbox{-} 2{\rm{\alpha }})]$. If OA is Regions I or IV, P is greater than 0, and the rectificated vector poses a negative effect to the performance of DOPC; but if OA is in Regions II or III, P is less than 0, and the rectification affects the performance of DOPC positively. Within or through a thick scattering medium where light is multiply-scattered, the phase profiles of the resultant optical field (i.e. speckle patterns) are randomly distributed, suggesting that the overall contributions of the positive and negative scenario cancel out. As a result, only the non-rectificated components function contributively to the PBR of DOPC. As known, with a full-phase modulation approach, the PBR of DOPC can be expressed by PBR ≈ πn/4, where *N* is the number of the controlled modes^25,36,37^. With the phase rectification-based DOPC proposed here, about half of the controlled modes have counteracted among themselves. Therefore, the theoretical PBR of this method is

7 $${{\rm{PBR}}}_{{\rm{rect}}}\approx {\rm{\pi }}N/{\rm{8}}$$

Based on Eqs (3–6), the obtainable PBR is numerically calculated for full phase, phase rectification, and binary phase modulations^38^, respectively, and is shown in Fig. 4(b). One can see the ratios between the PBR and the controlled number are 0.80, 0.40, and 0.31, respectively. Moreover, this simulated ratio for phase rectification-based DOPC is quite consistent with the theoretical value predicted from Eq. (7).

## Results

**Figure 5:**
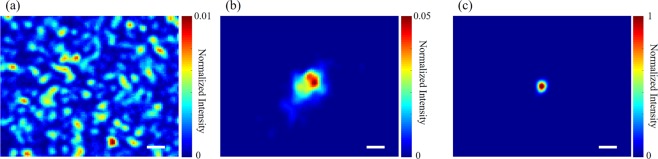
(**a**) The optical field recorded by Camera 2 when the SLM is displayed a random phase pattern, showing a random speckle pattern. (**b**) When the SLM was loaded with the optimized phase pattern before calibration, an optical focus was formed with a PBR of ~460. (**c**) When the SLM was loaded with the optimized phase pattern after calibration, the PBR of the optical focus can be up to ~23000. The scale bars represent 50 μm. The color bars in (**a**) and (**b**) are normalized to the peak intensity in (**c**).

Experiments were performed to validate the proposed DOPC setup. A ground glass (DG10–600, Thorlabs) was used as the scattering medium. When an optimized phase pattern was obtained and loaded on the SLM, an optical focus appeared as shown in Fig. 5b,c. Especially, with the proposed calibration and phase rectification, the PBR of the optical focus can go up to ~23,000 (Fig. 5c), and the focal spot is about 10 μm (FWHM) along both X and Y axes. Considering that the speckle size makes up about 20 pixels of the SLM and the number of independent control units on the SLM are 1920 × 1080, we estimate that the theoretical PBR is about 40,700. In contrast, we did not observe any focus when a random phase map was displayed on the SLM (Fig. 5a). Without the proposed SLM calibration, a spatially uneven optical focus achieved yet with a PBR of only ~460 and a FWHM dimension of 50 μm. Therefore, our method can improve the DOPC performance by 50 and 5 times regarding the PBR and the focal spot size, respectively. Moreover, the experimentally achieved PBR of 23,000 has reached ~56.5% of the theoretical one, which, to be best of our knowledge, approaches to the highest focusing efficiency (66% in ref. ^29^) for all DOPC experiments reported thus far.

**Figure 6:**
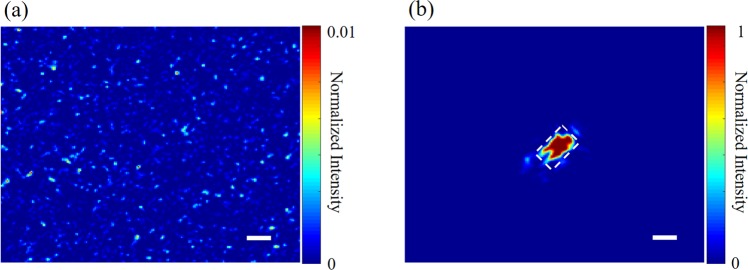
(**a**) The image of a metal chip recorded by Camera 2 when a random pattern was displayed on the SLM. (**b**) The image of the same metal chip when TRAP optimization was performed. The dashed frame in (**b**) contours the position and the shape of the metal chip. The scale bars represent 100 μm.

In order to test the ability of our system for optical focus inside scattering media, we carried out a set of time-reversed adapted-perturbation (TRAP) optical focusing experiment with a schematic setup similar to ref. ^25^. A small chip (silicon semiconductor chip coated by gold; 100 × 150 μm^2^) was tightly sandwiched between two microscope slides, forming an optical target attached to a translation platform. Two scattering layers were mounted before and after this optical target along the optical axis, serving as a scattering medium with a movable optical target inside. In experiment, the chip was first positioned outside the field of view, but later translated into the view, inducing perturbation to the optical fields recorded out of the scattering medium. With the TRAP procedure, a phase pattern that counteracts the turbidity of the scattering medium could be computed. When the phase pattern was displayed on the SLM during the playback process, diffused light could be refocused onto the chip, as seen in Fig. 6b, where the chip was clearly imaged with a PBR of 875. In comparison, when the SLM was displayed with a random pattern, a seemingly random speckle image, as shown in Fig. 6a, was obtained as light was multiply scattered within the scattering medium.

## Discussion

The calibration of the curvature of the SLM is a critical step towards reliable and high quality digital optical phase conjugation. Therefore, a few studies in the field have thus far dedicated to this topic. For example, in ref. ^29^, researchers used a Hadamard-pattern based iteration method to compensate for the curvature of the SLM and an extra auto-alignment method to fine tune the alignment between the SLM and the camera; a maximum PBR of 120,000 and a maximum efficiency of 66% were obtained. A bit later^30^, researchers used an orthonormal rectangular polynomials iteration method for SLM curvature compensation; a maximum PBR of 121,000 and a PBR improvement by a factor of 20 were obtained. No doubt both of the above-mentioned approaches can calibrate the curvature of the SLM effectively, especially when the number of independently controlled super-pixels for iteration on the SLM are large enough to represent as many major surface defects as possible. Note that, however, time consumed for iteration is directly proportional to the number of iterations (or the controlled units), while one single iteration takes about 1 second, mainly throttled by the slow refreshing rate of the SLM device. Therefore, one has to balance the calibration efficiency and the time consumption. It can be more demanding if the misalignment of the system or the disturbance to the system takes place during the experiment, which diminishes or even paralyzes the effect of the already-sought compensation. In this scenario, a new round of iterative compensation procedure is required. Therefore, in this paper, we introduce a plain DOPC setup that allows for rapid, reliable and high-fidelity optical phase conjugation, enabling effective optical focusing through or within scattering media. The four-phase calibration method, albeit seemingly unappealing, is actually quite pratical and convenient to be implemented, and it has not yet been reported in the context of digital optical phase conjugation. In our experiment, the calibration process, taking up only ~0.8 seconds, is embedded into the three-stage DOPC procedure. By doing so, the system misalignment or disturbance emerging or evolving in experiment can be inherently calibrated and compensated, ensuring a more robust performance over time. Moreover, benefiting from the non-iterative operation, the proposed method can achieve a full-pixel (1920 × 1080) compensation for the SLM curvature. This advantage allows for higher PBR improvement ratio when the SLM curvature and reference beam imperfection is calibrated: ours is 50 times as seen in Fig. 6, while it is only 2–5^29^ and 20^30^ times, respectively, in former studies. On the other hand, due to the lack of an EOM at hand, in this work, we used a phase rectification method, instead of the popularly used EOM-based full phase method, to retrieve the signal beam hologram information. As known, EOM is widely used for phase shifting in many scenarios, especially in the in-line DOPC system. It is necessary to clarify that the aim of the proposed phase rectification method is not to challenge this situation, but rather a substitute approach when an EOM is inaccessible. Lastly but not the least, while this article was under peer’s review, a similar SLM curvature compensation method in conjugation with autocovariance analysis and orthonormal rectangular polynomials^39^ was reported to improve the performance of DOPC. Nevertheless, the methods in the current article were independently conceived and developed. Moreover, such a curvature compensation method, integrated with the phase rectification operation, can serve as a relatively plain protocol to simplify the DOPC system as well as its operation and maintenance.

## Conclusion

In this paper, a plain self-embedded four-phase approach is developed to calibrate and compensate for the SLM surface curvature rapidly and effectively. A non-phase-shifting in-line holography approach is used to retrieve the phase information of the signal beam, generating effective optical focusing through or into scattering media. In experiment, the DOPC performance has been improved by 50 and 5 times, respectively, regarding the PBR and the spot size of the optical focus. Moreover, the focusing efficiency, as measured by the ratio between the experimental PBR to the theoretical one, is ~56.5%. Our setup provides a plain yet high-fidelity DOPC platform to enable effective optical focusing of diffused light. If further engineered, especially if the focusing time of the three-stage procedure can be reduced from the current several seconds down to the order of ms, it can potentially advance DOPC towards wide applications.
